# Binge Ethanol and MDMA Combination Exacerbates Toxic Cardiac Effects by Inducing Cellular Stress

**DOI:** 10.1371/journal.pone.0141502

**Published:** 2015-10-28

**Authors:** Javier Navarro-Zaragoza, Clara Ros-Simó, María-Victoria Milanés, Olga Valverde, María-Luisa Laorden

**Affiliations:** 1 Department of Pharmacology, Faculty of Medicine, University of Murcia, Murcia, Spain; 2 Grup de Recerca en Neurobiologia del Comportament (GRNC), Universitat Pompeu Fabra, Barcelona, Spain; University of Kentucky, UNITED STATES

## Abstract

Binge drinking is a common pattern of ethanol consumption among young people. Binge drinkers are especially susceptible to brain damage when other substances are co-administered, in particular 3,4 methylendioxymethamphetamine (MDMA). The aim of the present work was to study the mechanisms implicated in the adaptive changes observed after administration of these drugs of abuse. So, we have evaluated the cardiac sympathetic activity and the expression and activation of heat shock protein 27 (HSP27), after voluntary binge ethanol consumption, alone and in combination with MDMA. Both parameters are markers of stressful situations and they could be modified inducing several alterations in different systems. Adolescent mice received MDMA, ethanol or both (ethanol plus MDMA). Drinking in the dark (DID) procedure was used as a model of binge. Noradrenaline (NA) turnover, tyrosine hydroxylase (TH), TH phosphorylated at serine 31 and HSP27 expression and its phosphorylation at serine 82 were evaluated in adolescent mice 48 h, 72 h, and 7 days after treatments in the left ventricle. NA and normetanephrine (NMN) were determined by high-performance liquid chromatography (HPLC); TH and HSP27 expression and phosphorylation were measured by quantitative blot immunollabeling using specific antibodies. Ethanol and MDMA co-administration increased NA turnover and TH expression and phosphorylation versus the consumption of each one of these drugs. In parallel with the described modifications in the cardiac sympathetic activity, our results showed that binge ethanol+MDMA exposure is associated with an increase in HSP27 expression and phosphorylation in the left ventricle, supporting the idea that the combination of both drugs exacerbates the cellular stress induced by ethanol or MDMA alone.

## Introduction

Binge is a pattern of alcohol (ethanol) consumption that results in 0.08 g per cent or above alcohol concentration in blood, i.e. 5 or more drinks in a single occasion [1]. Most of clinical, histopathological, and biochemical studies consider the effect of chronic alcohol intoxication on myocardial injury. However, much less attention has been paid to acute alcohol (binge drinking)-induced cardiotoxicity, even though alcohol binging is much more common than alcohol dependence (for review see [2,3]). Concerning to binge drinking, the literature has demonstrated transient myocardial subtle changes in cardiac magnetic resonance, accompanied by increase in serological marker of myocardial injury [4] and pathological sequelae including disruption of myofibrillary architecture and finally compromised myocardial contractility function [5–7].

3,4-Methylenedioxy-N-methamphetamine (MDMA, ecstasy) is an amphetamine derivative and is a popular type of drug that is abused due to its effects on the central nervous system (CNS), including alertness and euphoria. Apart from its desired effects on mood and perception, MDMA is a potent stimulant of cardiovascular action increasing heart rate and blood pressure [8–10]. There are also experimental and clinical data which document that MDMA can alter cardiovascular function and produce cardiac toxicity, including rhythm disturbances, infarction and sudden death (for review see [11]). Moreover, ecstasy users are generally multi-drug users and alcoholic beverages are commonly combined with MDMA [12]. The co-abuse of alcohol and MDMA is prevalent worldwide [13–14].

Although the combined consumption of alcohol and MDMA in humans is not fully understood, some explanations have been proposed. Among them, is the fact that the combined intake of alcohol and MDMA produces a longer lasting euphoria and feelings of well being, than the consumption of either drug alone, suggesting an increased subjective perception of the positive effects induced by the drug [15]. Also, it has been proposed that ethanol attenuates the negative side effects of MDMA, in particular, regarding the hyperthermia associated to MDMA intake [16].

The extensive co-abuse of ethanol and MDMA is of particular concern since studies indicate that the co-abuse of ethanol and MDMA increases the risk of organ damage. For example, the co-administration of ethanol with MDMA enhanced MDMA-mediated long term neurotoxicity [17] as well as hepatotoxicity [18–19]. However, the cardiac effects of this common combination are not well established. As far as we know the combination of binge ethanol and MDMA on cardiac tissues has not been assessed previously.

Drugs of abuse appear to activate the hypothalamic-pituitary-adrenocortical (HPA) axis and thus to able to induce considerable stress response in both experimental and clinical situations [20–21]. Besides, exposure to a stressful situation leads to the activation of the cathecolaminergic system. Activation of this pathway can damage the heart [22]. Furthermore, the profound cellular stress induced by drugs of abuse is also evidenced by the overexpression of heat shock proteins (HSPs) as well as massive alterations in different functions [23]. HSPs were identified primarily on the basis of their fast and typically protective response to cellular stressors. HSPs are rapidly induced at the transcriptional level after stress, but also undergo several post-translational modifications that alter their functional roles for use as immediate response elements [24].

In this study, a daily limited-access alcohol intake model named drinking in the dark (DID) [25] was used as a model to assess the cardiac changes observed in binge drinkers. We have used CD1 mice,a strain with low alcohol prevalence. The ethanol preference differences between CD1 and other strains have been atributed to changes in ethanol metabolism and palatability [26]. The work presented here evaluates, first, the effects of voluntary binge ethanol intake or MDMA administration on the cardiac sympathetic pathways by measuring noradrenaline (NA), its peripheral metabolite (NMN), and tyrosine hydroxylase (TH), rate-limiting enzyme in catecholamine synthesis. On the other hand, we have analyzed the severity of cellular stress caused by ethanol or MDMA by investigating the expression of HSP27, a member of the small HSP family which is highly expressed in the heart [27]. Second, it measures the effects of acute co-administration of MDMA on cardiac changes induced by binge ethanol. The performance of these experiments allow us to establish a relation between binge ethanol plus MDMA administration and sympathetic cardiac activity.

## Methods

### Subjects

Adolescent naive male CD-1 mice (postnatal day 21) weighing 25–30 g at the beginning of the experiments were used in this study. Mice were purchased from Charles River (France) and housed four per cage during 7 days (quarantine period) until 1 week prior to the beginning of experiments when mice were individually housed (postnatal day 28). Experiments started 7 days after the individualization (postnatal day 36). Animal rooms were controlled for temperature (22±1°C), humidity (55±10%) and photo-period (12:12 L/D). Lights were turned on at 08:00 hours and off at 20:00 hours. One week prior to the experiment, mice were switched to a reverse light/dark schedule in which lights turned on at 19:00 hours and off at 07:00 hours. Food and water were available ad libitum except when water was substituted for ethanol for 2 or 4 h per day according to DID procedure, described below. All the animals care and experimental procedures were conducted according to the guidelines of the European Communities Directive 2010/63/EU regulating animal research and were approved by the local ethical committee "Comité Ético de Experimentación Animal del Parc de Recerca Biomedica de Barcelona" (CEEA-PRBB).

### Drugs and reagents

Racemic MDMA hydrochloride was purchased from Lipomed, A.G. (Arlesheim, Switzerland), dissolved in 0.9% physiological saline in order to obtain a dose of 20 mg/kg (2 mg/ml) expressed as the salt, and injected in a volume of 0.1 ml/10 g body weight by intraperitoneal (i.p.) route of administration. Ethyl alcohol was purchased from Merck Chemicals (Darmstadt, Germany) and diluted in tap water in order to obtain a 20% (v/v) ethanol solution. Sodium dodecylsulphate, polyacrylamide gel and poly vinylidene difluoride (PVDF) membrane were obtained from Bio-Rad Laboratory (Teknovas, Bilbao, Spain). Reagents: proteases inhibitor (Boehringer Mannheim, Germany); phosphatase inhibitor cocktail set (Cabiochem, German). High-performance liquid chromatography (HPLC) reagents were purchased from Sigma Aldrich (San Luis, MO, USA).

### Drinking in the dark procedure

**Fig 1:**
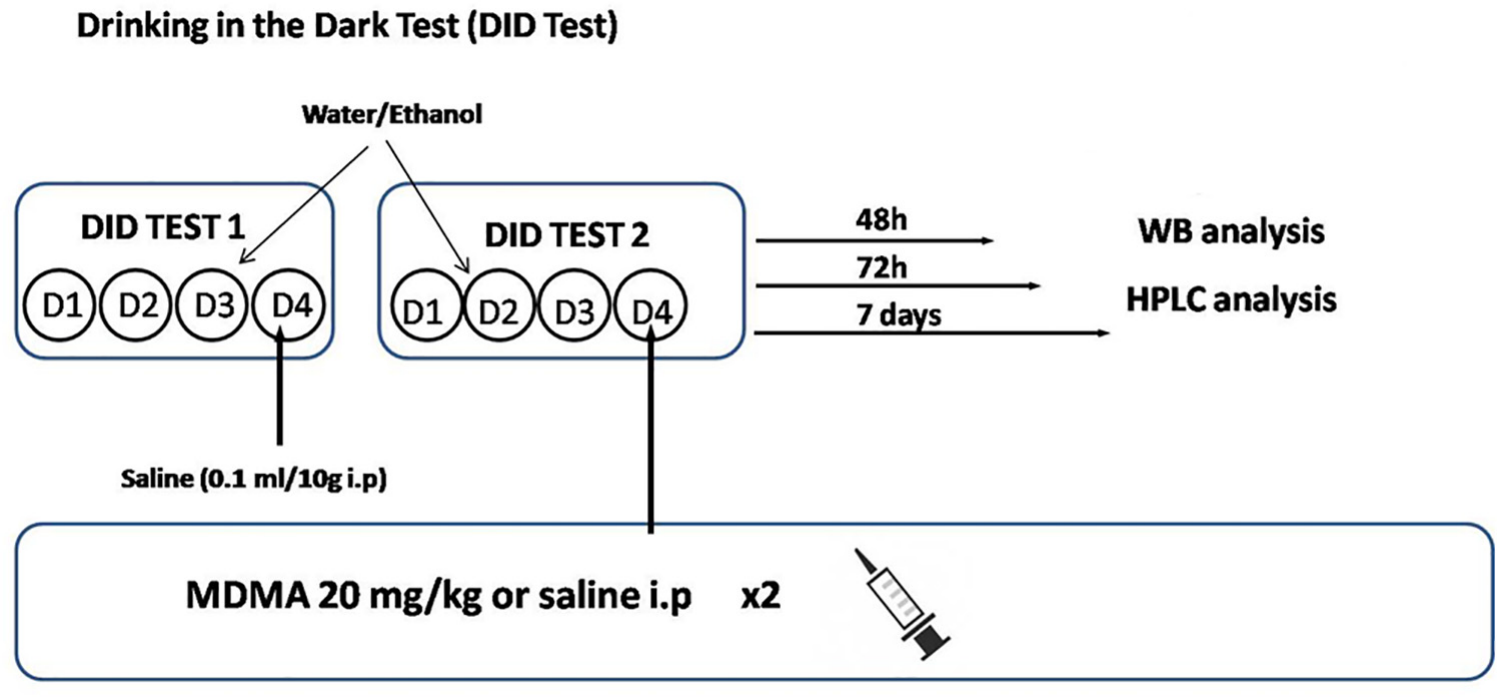
Drinking in the dark test (DID test) (see Methods section).

This procedure was conducted as previously reported [25]. Briefly, food was removed and the water bottles were replaced with 10-ml graduated cylinders fitted with sipper tubes containing either 20% (v/v) ethanol in tap water or only tap water (groups Ethanol and Water, respectively) 3 h after lights were turned off in the animal rooms. During this time, animals were maintained in home cages individually housed (see above). The ethanol or water cylinders remained in place for 2 h. After the 2-h period, individual intake was recorded and food and water bottles were replaced. This procedure was repeated on days 2 and 3 and fresh fluids were provided each day. On day 4, all subjects were injected with a single dose of saline (0.1 ml/10 g, i.p.) prior to the DID procedure. On the following week, the DID procedure was conducted again as described above. On day 4 subjects were injected by a single dose of MDMA (20 mg/kg) or saline (i.p). At this day, the ethanol or water cylinders were left for 4 h and intakes were recorded after 2 and 4 h respectively. After the first recording (2h), the animals received a second injection of MDMA (20 mg/kg) or saline (i.p.) (Fig 1). The dose of MDMA was selected in accordance to previous studies [28–30]. Following the 4 h free access to fluid and immediately after recording fluid intake, ethanol and water cylinders were replaced with water bottles. Then, animals were separated in three groups: 48 h (postnatal day 40), 72 h (postnatal day 41) or 7 days (postnatal day 45) after the last MDMA or saline injection (under ethanol absence) (Fig 1). Mice were sacrificed to measure NA, NMN, TH, TH phosphorylated at serine 31, HSP27 and HSP27 phosphorylated at serine 82 in the left ventricle.

### Determination of noradrenaline (NA) and its metabolite normetanephrine (NMN) in the left ventricle

NA and NMN were determined by HPLC with electrochemical detection. Each tissue was weighted, placed in a dry-cooled propylene vial and homogenized. The homogenates were centrifuged (10000 g, 4°C), the supernatant layer was removed into a 1-ml syringe and filtered through a 0.45 μm filter (Millipore, Bedford, USA) and centrifuged (6000 g, 4°C) again through Ultra free MC 0.2 μm filter (Millipore). From each sample, 10 μl were injected into a 5-μm C18 reverse phase column (Waters, Milford, MA, USA) through a 200 μl loop Rheodyne syringe-loading injector. The mobile phase consisted of a 95% (v/v) mixture of water and methanol with sodium acetate (50 mM), citric acid (20 mM), L-octyl-sodium sulfonate (3.75 mM), di-n-butylamine (1 mM) and EDTA (0.135 mM), adjusted to pH 4.3. Chromatographic data were analysed with Millenium 2010 Chromatography Manager Equipment (Millipore). NA and its metabolite were simultaneously detected and quantified by reference to calibration curves run at the beginning of the assays. The content of NA and NMN in the left ventricle was expressed as nanogram per gram of tissue weight.

### Western Blotting

Samples were placed in homogenization buffer [phosphate buffered saline, 2% sodium dodecylsulfate (SDS) plus protease inhibitors (Roche, Germany) and phosphatase inhibitors Cocktail Set (Calbiochem, Germany)], homogenized and centrifuged at 6000 g at 4°C. Equal amounts of protein (50 μg/lane) from each sample were loaded on a 10% SDS-polyacrylamide gel (SDS-PAGE), electrophoresed, and transferred onto a PVDF membrane using a Mini Trans-Blot Electrophoresis Transfer Cell (Bio-Rad Lab., California, USA). Non-specific binding of antibodies was prevented by incubating membranes with 1% bovine serum albumin (BSA) in tris buffer saline tween (TBST: 10 mM Tris-HCl, pH 7.6, 150 mM NaCl, 0.05% Tween 20). Blots were incubated overnight with the following primary anti-rabbit antibodies: anti- polyclonal anti-phospho serine 31 TH, polyclonal anti-total TH (1:500 dilution, Millipore, Temecula, CA, USA), and polyclonal anti-phospho Ser82 HSP27 (1:500; Santa Cruz Biotecnology, Santa Cruz, CA, USA) and goat antibody polyclonal anti-total HSP27 antibody (1:750; Santa Cruz Biotecnology, Santa Cruz, CA, USA) in TBST with BSA. Following extensive washings with TBST, the membranes were incubated for 1 h with peroxidase-labeled secondary antibodies at room temperature. After washing, immunoreactivity was detected with an enhanced chemiluminescent/chemifluorescent western blot detection system (ECL Plus, GE Healthcare, UK) and visualized by a Typhoon 9410 variable mode Imager (GE Healthcare). Antibodies were stripped from the blots by incubation with stripping buffer (glycine 25mM and SDS 1%, pH2), for 1 h at 37°C. We used anti α-tubulin (Cell Signaling Technology, Danvers, MA, USA; 52 kDa) as our loading control for all the experiments. Quantification of immunoreactivity bands corresponding to total TH (62 kDa), TH phosphorylated at serine 31 (45 kDa), total HSP27, and phosphoHSP27 (27 kDa) was carried out by densitometry (AlphaImager, Nucliber, Madrid). Experimental and control samples were included in the same blots and relative variations between bands were calculated in the same image.

### Statistical analysis

Data are expressed as mean ± SEM. Statistical analysis was determined by two-way analysis of variance (ANOVA). The Newman-Keuls test was used as a post-hoc whenever a significant difference between three or more sample means was revealed by an analysis of variance (ANOVA). Differences with a p<0.05 were considered significant.

## Results

Water and ethanol consumption was measured for each mouse everyday during the DID procedure (Fig 1). One-way ANOVA for water consumption did not show differences in the total fluid (ml) consumed between water-treated groups (Water × MDMA vs. Water × Saline). Regarding the amount of ethanol (g EtOH/kg and ml) consumed, no differences were found between groups (Ethanol × MDMA vs. Ethanol × Saline) in none of the days of the DID procedure. Thus, consumption of water and ethanol remain consistent across days in each group. In addition, this procedure has been shown to produce consistent blood ethanol concentrations (BECs), in our case BEC obtained was 62,6 + 11,49 mg/dL.

### Ethanol binge drinking and MDMA effects on NA, NMN content and NA turnover

**Fig 2:**
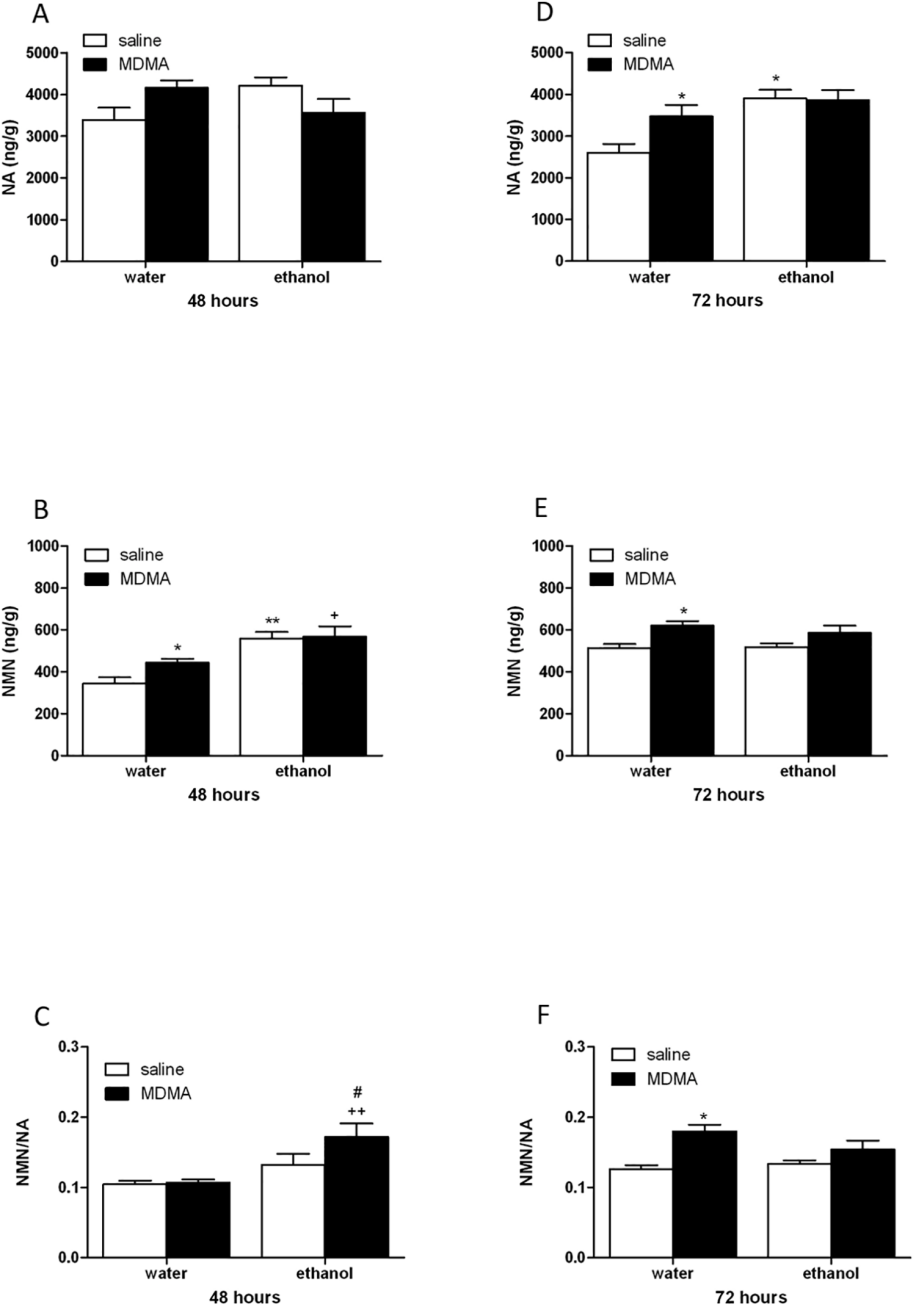
Noradenaline(NA, A,D) and normetanephrine (NMN B,E,) content and NMN/NA ratio (C,F) in the left ventricle from animal treated with water or ethanol and sacrificed 48 or 72 h after the last injection of MDMA or saline. Data are the mean±SEM (n = 5). *p<0.05, **p<0.01 versus water+saline; +p<0.05, ++p<0.01 versus water+MDMA; #p<0.05 versus ethanol+saline.

Two-way ANOVA for NA 48 h after MDMA or binge ethanol exposure revealed no effect of ethanol pretreatment [F(1,14) = 0,21; *P* = 0.6570], no significant effect of acute treatment [F(1,14) = 0,07; *P* = 0.8009], and a significant interaction between ethanol pretreatment and MDMA administration [F(1,14) = 8,21; *P* = 0.0125]. As shown in Fig 2A Newman Keuls post-hoc analysis did not show significant differences 48 h after MDMA or binge ethanol exposure. In contrast, two-way ANOVA for NMN revealed a significant effect of ethanol pretreatment [F(1,14) = 26,41; *P* = 0.0002], no significant effect of MDMA treatment [F(1,14) = 2,73; *P* = 0.1208], and no significant interaction between ethanol pretreatment and MDMA administration [F(1,14) = 1,95; *P* = 0.1848]. However, post-hoc test revealed that MDMA or binge ethanol produced a significant (p<0.05, p<0.01) increase in NMN content when compared with control group (water+saline). Ethanol and MDMA-treated animals (ethanol+MDMA) also showed a significant (p<0.05) enhancement of NMN versus the group treated with water+MDMA (Fig 2B). In regard to NMN/NA ratio, an index of NA turnover, two-way ANOVA showed a significant effect of ethanol pretreatment [F(1,14) = 13,55; *P* = 0.0025], no significant effect of acute treatment [F(1,14) = 2,82; *P* = 0.1152], and no significant interaction between ethanol pretreatment and MDMA administration [F(1,14) = 2,15; *P* = 0.1643]. Newman-Keuls indicated an increased NA turnover in the group treated with ethanol+MDMA when compared with ethanol+saline group (p<0.05) and water+MDMA group (p<0.01) (Fig 2C).

Seventy-two hours after last MDMA injection we have observed for NA a significant effect of ethanol pretreatment [F(1,13) = 11,93; *P* = 0.0043], no significant effect of acute treatment [F(1,13) = 2,83; *P* = 0.1161], and no significant interaction between ethanol pretreatment and MDMA administration [F(1,13) = 3,49; *P* = 0.0844]. Therefore, Newman-Keuls test showed an increase (p<0.05) in NA content in the MDMA or binge ethanol-treated mice compared to control group. The increase of NA content was not observed in mice pre-exposed to binge ethanol and treated with MDMA (Fig 2D). Results for NMN content after two-way ANOVA analysis showed no significant effect of ethanol pretreatment [F(1,13) = 0,34; *P* = 0.5685], a significant effect of MDMA treatment [F(1,13) = 12,22; *P* = 0.0039], and no significant interaction between ethanol pretreatment and MDMA administration [F(1,13) = 0,62; *P* = 0.4441]. Moreover, Newman-Keuls for NMN content showed that there was a significant (p<0.05) increase in the group treated with MDMA (Fig 2E). Two-way analysis for NA turnover 72 hours after MDMA or saline administration resulted in no significant effect of ethanol pretreatment [F(1,13) = 1,04; *P* = 0.3270], a significant effect of acute treatment [F(1,13) = 16,97; *P* = 0.0012], and no significant interaction between ethanol pretreatment and MDMA administration [F(1,13) = 3,41; *P* = 0.0875]. Newman-Keuls for NA turnover resulted in a significant (p<0.05) increase in the group treated with MDMA (Fig 2F). Altogether, these data suggested an increased sympathetic cardiac activity after 48 or 72 h binge ethanol+MDMA administration.

### Ethanol binge drinking and MDMA effects on TH and TH phosphorylated at serine 31

**Fig 3:**
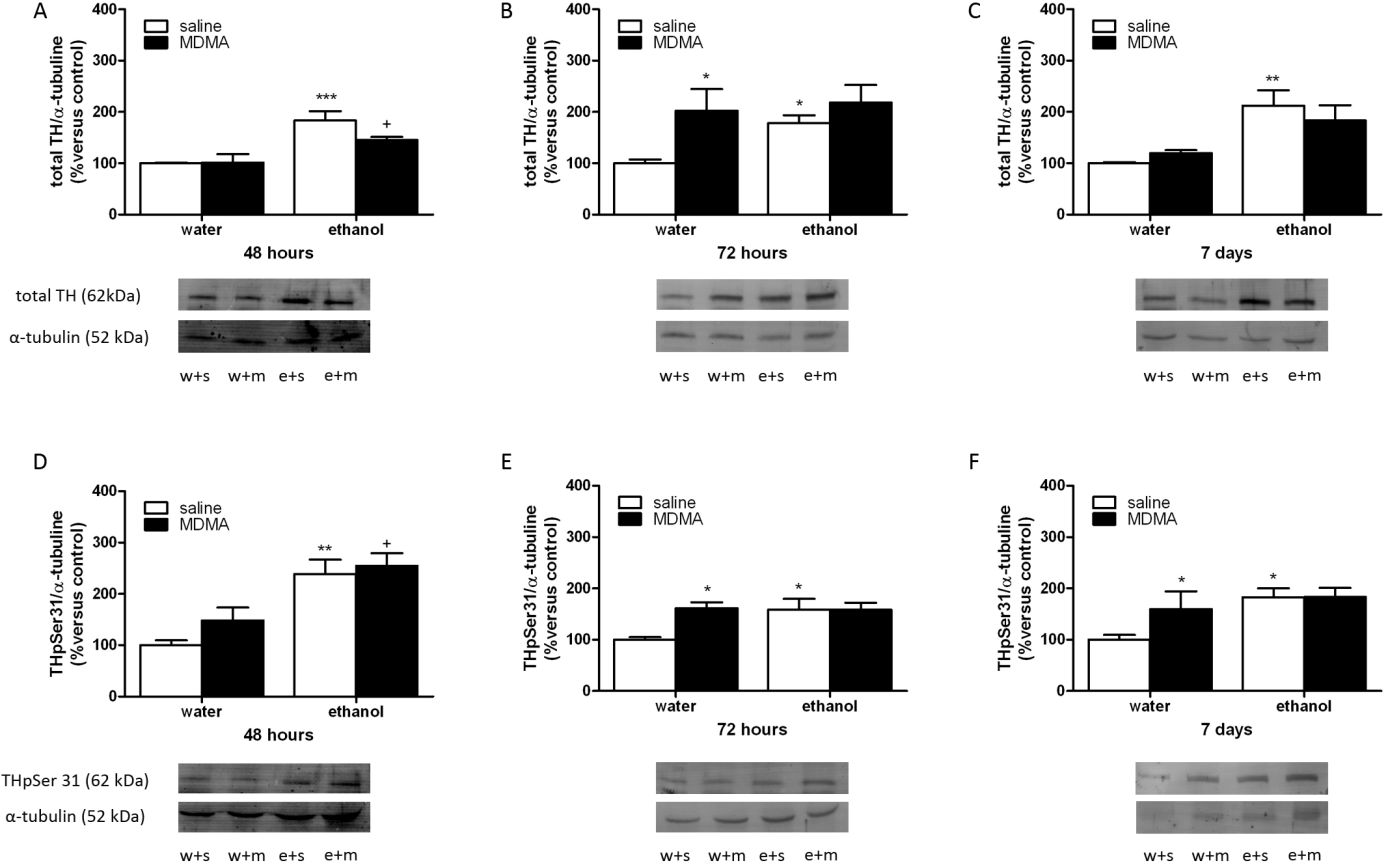
Western-blotting analysis of Total TH (A,B,C) and TH phosphorylated (p) at serine 31 (Ser 31) (D,E,F) in the left ventricle from animal treated with water (w) or etahanol (e) and sacrificed 48h, 72h or 7 days after the last injection of MDMA (m) or saline (s). The immunoreactivity corresponding to total TH or TH pSer31 is expressed as a percentage of that in the control group defined as 100% value. Data are the mean±SEM (n = 5–6). *p<0.05,**p < 0.01, ***p<0.001 versus water+saline; +p<0.05 versus water+MDMA.

The influence of MDMA and binge ethanol on the immunoreactivity of total TH was examined in the left ventricle 48 h, 72 h and 7 days after the last MDMA or saline injection (Fig 3A, 3B and 3C). Two-way ANOVA analysis for total TH 48 hours after MDMA or saline injection revealed a significant effect of ethanol pretreatment [F(1,14) = 6,37; *P* = 0.0243], no significant effect of acute treatment [F(1,14) = 0,04; *P* = 0.8378], and no significant interaction between ethanol pretreatment and MDMA administration [F(1,14) = 0,09; *P* = 0.7745]. This analysis showed in contrast no significant effect of ethanol pretreatment 72 hours after MDMA administration [F(1,16) = 4,41; *P* = 0.0520], a significant effect of MDMA treatment [F(1,16) = 9,70; *P* = 0.0067], and no significant interaction between ethanol pretreatment and MDMA administration [F(1,16) = 1,82; *P* = 0.1956]. Finally two-way for 7 days group revealed a significant effect of ethanol pretreatment [F(1,15) = 15,46; *P* = 0.0015], no significant effect of acute treatment [F(1,15) = 0,04; *P* = 0.8439], and no significant interaction between ethanol pretreatment and MDMA administration [F(1,15) = 1,15; *P* = 0.3013]. Newman-Keuls showed that total TH levels were unchanged 48 h or 7 days after MDMA treatment, whereas these levels were increased (p<0.05) 72 h after the treatment. Animals exposed to binge ethanol present an increased total TH levels 48 h (p<0.001), 72 h (p<0.05) or 7 days (p<0.01) after its exposure. In addition, there is an increased total TH levels 48 h after MDMA+ethanol versus MDMA alone (Fig 3A, 3B and 3C)

Additionally experiments were performed in the left ventricle to determine whether MDMA or binge ethanol would activate phosphorylation of TH at serine 31 at different time points (Fig 3A, 3B and 3C). Two-way ANOVA analysis for TH at serine 31 48 hours after MDMA or saline injection revealed a significant effect of ethanol pretreatment [F(1,13) = 25,72; *P* = 0.0002], no significant effect of acute treatment [F(1,13) = 1,79; *P* = 0.2037], and no significant interaction between ethanol pretreatment and MDMA administration [F(1,13) = 0,44; *P* = 0.5202]. This analysis showed in contrast no significant effect of ethanol pretreatment 72 hours after MDMA administration [F(1,15) = 3,69; *P* = 0.0738], a significant effect of MDMA treatment [F(1,15) = 4,50; *P* = 0.0510], and a significant interaction between ethanol pretreatment and MDMA administration [F(1,15) = 4,71; *P* = 0.0465]. Finally two-way for 7 days group of treatment revealed a significant effect of ethanol pretreatment [F(1,16) = 7,54; *P* = 0.0144], no significant effect of acute treatment [F(1,16) = 2,45; *P* = 0.1371], and no significant interaction between ethanol pretreatment and MDMA administration [F(1,16) = 2,31; *P* = 0.1481]. Newman-Keuls post-hoc test demonstrated that 72 h (p<0.05) and 7 days (p<0.05) after MDMA treatment or binge ethanol exposure there were an increased phosphorylation of TH at serine 31 (Fig 3E and 3F). After 48 h, binge ethanol exposure also increased (p<0.01) TH phosphorylated at serine 31 but there were not changed in MDMA group (Fig 3D). There was a significant (p<0.05) interaction between ethanol+MDMA 48 h after their administration when compared to MDMA alone (Fig 3D). The last experiments show that the increase in sympathetic cardiac activity observed in our study was induced by an enhancement of TH phosphorylation.

### Expression of HSP27 and phospho-HSP27 after ethanol binge drinking or MDMA treatment

**Fig 4:**
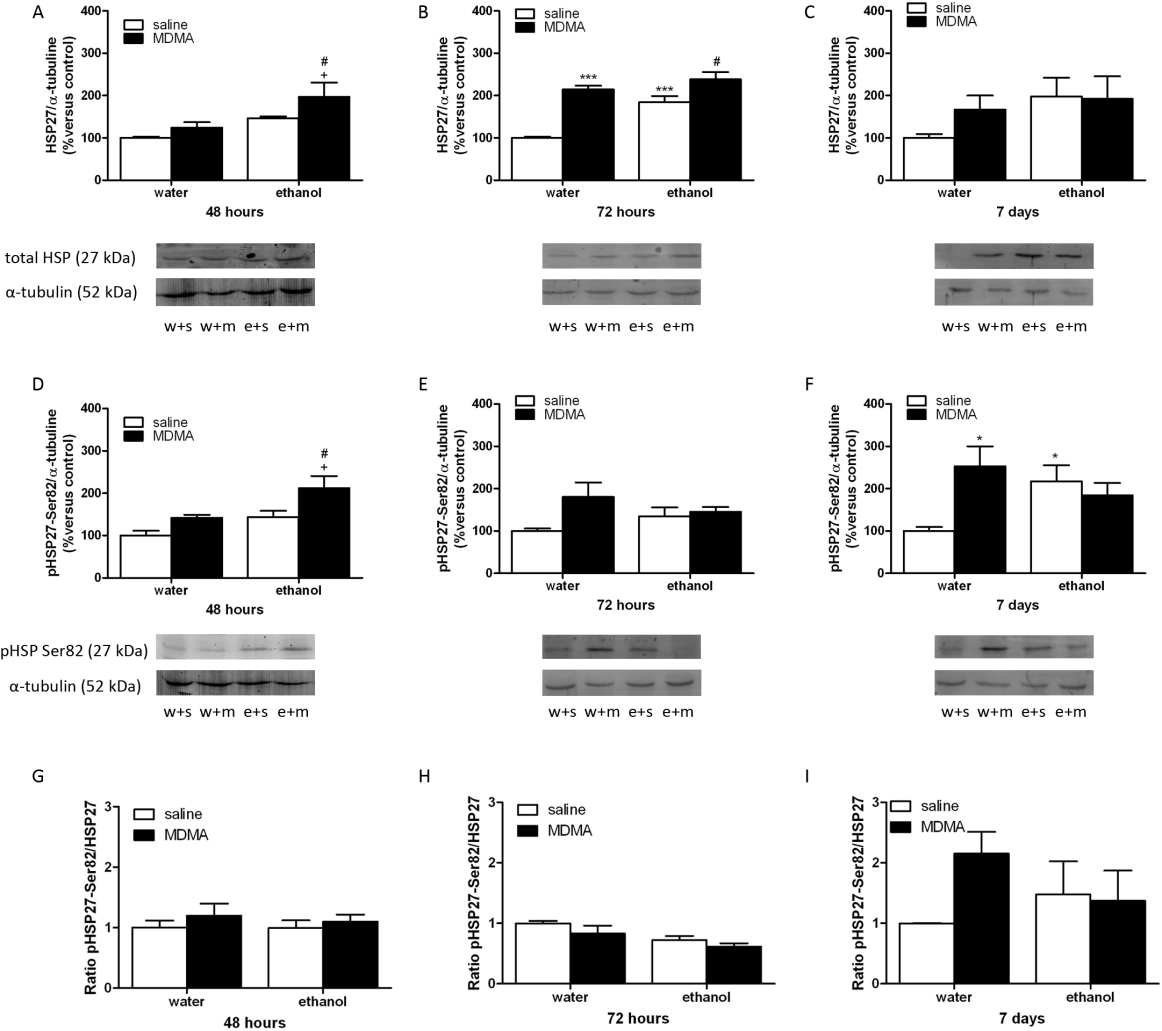
Western-blotting analysis of HSP27 (A,B,C), HSP27 phosphorylated (p) at serine 82 (Ser82) (D,E,F) and pHSP27/HSP27 ratio (G,H,I) in the left ventricle from animals treated with water (w) or ethanol (e) and sacrificed 48h, 72h or 7 days after the last injection of MDMA (m) or saline (s). The immunoreactivity corresponding to HSP27 or HSP27pSer82 is expressed as a percentage of that in the control group defined as 100% value. Data are the mean±SEM (n = 5). *p<0.05,***p<0.001 versus water+saline; +p<0.05 versus water+MDMA; #p<0.05 versus ethanol+saline.

We examined HSP27 expression and phospho-HSP27 at serine 82 to determine the magnitude and severity of cellular stress during binge ethanol exposure or MDMA treatment. Two-way ANOVA analysis for HSP 27 48 hours after MDMA or saline injection revealed a significant effect of ethanol pretreatment [F(1,13) = 12,23; *P* = 0.0039], a significant effect of acute treatment [F(1,13) = 4,85; *P* = 0.0463], but no significant interaction between ethanol pretreatment and MDMA administration [F(1,13) = 0,60; *P* = 0.4520]. This analysis also showed a significant effect of ethanol pretreatment 72 hours after MDMA administration [F(1,13) = 17,02; *P* = 0.0012], a significant effect of MDMA treatment [F(1,13) = 41,53; *P <* 0.0001], and a significant interaction between ethanol pretreatment and MDMA administration [F(1,13) = 5,27; *P* = 0.0389]. Finally two-way ANOVA for 7 days after MDMA administration group revealed no significant effect of ethanol pretreatment [F(1,14) = 2,11; *P* = 0.1679], no significant effect of acute treatment [F(1,14) = 0,50; *P* = 0.4893], and no significant interaction between ethanol pretreatment and MDMA administration [F(1,14) = 0,73; *P* = 0.4061]. Newman-Keuls post-hoc test revealed that HSP27 expression was significantly increased (p<0.001) 72 h after ethanol exposure or MDMA treatment (Fig 4B). In addition, we have observed an increased (p<0.05) expression of HSP27 in ethanol+MDMA group versus ethanol or MDMA alone (Fig 4A), this interaction was also significant (p<0.05) versus ethanol alone 72 h after its exposure (Fig 4B). There were not changes in HSP 27 seven days after treatment (Fig 4C).

We also studied the phosphorylation of HSP27 at serine 82 in the left ventricle at different time points. Two-way ANOVA analysis for total TH 48 hours after MDMA or saline injection revealed a significant effect of ethanol pretreatment [F(1,14) = 8,98; *P* = 0.0096], a significant effect of acute treatment [F(1,14) = 8,48; *P* = 0.0114], and no significant interaction between ethanol pretreatment and MDMA administration [F(1,14) = 0,50; *P* = 0.4892]. This analysis also showed in contrast no significant effect of ethanol pretreatment 72 hours after MDMA administration [F(1,14) = 0.00; *P* = 0.9885], a significant effect of MDMA treatment [F(1,14) = 4,95; *P* = 0.0431], and no significant interaction between ethanol pretreatment and MDMA administration [F(1,14) = 2,97; *P* = 0.1071]. Finally two-way ANOVA for 7 days group revealed no significant effect of ethanol pretreatment [F(1,14) = 0,52; *P* = 0.4831], no significant effect of acute treatment [F(1,14) = 3,02; *P* = 0.1040], and a significant interaction between ethanol pretreatment and MDMA administration [F(1,14) = 7,27; *P* = 0.0174]. Finally, as shown in Fig 4D, Newman-Keuls post-hoc test showed a significant increased phosphorylation of HSP27 48 h after the MDMA injection in animals exposed to binge ethanol versus the group treated with binge ethanol or MDMA alone. There were no changes in any of the groups studied 72 h after the last MDMA injection (Fig 4E). Surprisingly, HSP27 expression was increased (p<0.05) seven days after binge exposure or MDMA-treatment versus the group that received water+saline, suggesting that both drugs of abuse induce long-term changes at heart level. The ratio phospho-HSP27-total HSP is represented in Fig 4G, 4H and 4I. As can be seen in this figure there were no differences between the groups studied 48 h, 72 h or 7 days after the last injection of MDMA.

## Discussion

The present study attempts to elucidate the cardiac effects of a binge pattern of ethanol consumption, and the influence of MDMA co-administration. The principal findings of the present study are as follows: 1) Ethanol binge drinking or MDMA administration alone increased NMN content in parallel with an enhancement in the expression of TH and TH phosphorylated at serine 31 at different time points, indicating an increased cardiac sympathetic activity, 2) The severity of cellular stress during ethanol binge or MDMA administration is also evidenced by the expression and activation (phosphorylation) of HSP27 and 3) NA turnover, total TH and HSP27 expression and phosphorylation were significantly higher after the combination of both drugs, indicating that these combination could produce a profound cellular stress that would damage the heart. We performed two-way ANOVA analysis due to the fact that two independent variables were involved in this experiment and we wanted to find the interaction between them.

Some of the effects described above can be observed even seven days after treatment, suggesting possible long-term effects of the treatment which may favour future compulsive use of the drugs [31]. CD1 mice were selected for our experiments due to the fact that this strain is considered to have low preference for alcohol [25,32]. The ethanol preference differences between CD1 and other strains such as C57BL/6J, have been attributed to produce changes in ethanol metabolism and palatability [26]). In any case, CD1 animals reach a blood ethanol concentration of about 50–70 mg%; the National Institute on alcohol abuse and alcoholism considers a blood ethanol concentration of 80 mg% as intoxication in humans [33].

Although a number of scenarios have been postulated with regard to the onset and progression of ethanol-induced myopathic changes, the precise mechanisms underlying alcohol-elicited cardiac anomalies remains elusive. It is known that binge drinking has adverse consequences on cardiovascular physiology and thrombosis/fibrolysis processes [34]. Single heavy drinking episodes (binge drinking) increase blood pressure, heart rate, acute coronary vasoconstriction, and ischemia (for review see [3]). Ethanol and MDMA are frequently co-abused [13,14,16,19,35] and evidence supports a drug interaction between these two agents [17–18]. It is important that we understand the mechanism by which MDMA interact to cause tissue damage in order to develop therapeutics or interventions measures to minimize damage. Present results demonstrated an increase NA and NMM content at different time point after the last MDMA or saline injection in ethanol or MDMA group. Our study also reveals an increased NA turnover in ethanol+MDMA-treated animals compared to ethanol- or MDMA-only-treated groups. In parallel with the changes observed in NMN/NA ratio we have evidenced an enhancement in the total TH and TH phosphorylated at serine 31 in ethanol or MDMA group at different time point. In addition, the association of ethanol and MDMA induced an increase TH expression and phosphorylation versus MDMA-only-treated group suggesting a greater increase in cardiac sympathetic pathways when both drugs are given associated. Changes in the state of phosphorylation of TH, the rate-limiting enzyme in the synthesis of catecholamines, are critically involved in the regulation of catecholamine synthesis and function. In particular, increases in the phosphorylation of serine 31 and serine 40 accelerate TH activity, thereby stimulating production of neurotransmitters in catecholamine terminals and then their release (for review, see [36–38]). Using phosphorylation state-specific antibody directed towards serine 31 phosphorylation of TH, present study showed that ethanol+MDMA exposure increased the levels of TH phosphorylated at serine 31 in the left ventricle concomitantly with the above described enhanced of NA turnover. Together this data provide evidence for TH phosphorylation after ethanol or MDMA exposure in the noradrenergic nerve terminals innervating the left ventricle and suggest an increased activity of cardiac sympathetic pathways which could be responsible of the cardiac toxicity induced by both drugs of abuse [39–43]. Furthermore, it has been proposed that drugs that perturb catecholaminergic function induce changes in THmRNA and protein expression [44–45]. It is known that TH protein levels and activity can be regulated by two different categories: short-term regulation of enzyme activity and medium-to long term regulation of gene expression (transcriptional regulation) [36]. Our data revealed an increased expression of TH and TH phosphorylated at serine 31 at different time points suggesting that transcriptional and post-transcriptional mechanisms could be, in part the molecular mechanisms involved in the activation of cardiac noradrenergic systems after ethanol and MDMA exposure or after the combination of both drugs.

It is well known that ethanol or MDMA cause oxidative/nitrosative stress and tissue injury in many organs including liver, brain, pancreas and heart [6,46–49]. Although potentiation of MDMA-mediated organ damage by ethanol it has been described [49], the mechanism of this boosting is poorly understood. The present investigation shows, for the first time, that binge ethanol+MDMA exposure is associated with an increase in the HSP27 expression and phosphorylation in the left ventricle, supporting the idea that the combination of both drugs exacerbates the cellular stress induced by ethanol or MDMA alone. This stress situation can produce ischemia and/or alteration in cellular membrane function [50], which contribute to tissue damage in many tissues and deaths in some cases [49]. Several studies [51–53] showed that HSP27 had a protective effect in ischemia/reperfusion animal models where oxidative stress was an important factor in neuronal cell death. Transduction with HSP27 can also increase viability of neuronal cells treated with hydrogen peroxide [52]. These studies indicate that HSP27 can attenuate the oxidative stress induced cell death.

According to previous data obtained in rats treated chronically with morphine, another drug of abuse [54–56], present results demonstrated an increase in HSP27 expression and phosphorylation in mice exposed to ethanol or MDMA; these effects were potentiated when the combination of both drugs was used. While HSP27 can block actin polymerization, the phosphorylation of HSP27 is related to re-organization of the actin-based cytoskeletal structures [57]. It has been suggested that this re-organization of the actin cytoskeleton induced by phosphorylation of HSP27 could lead to cytoprotection due to stabilization of actin filaments [58]. Altogether, these results support the idea that binge ethanol+MDMA induces profound cellular stress that could produce myocardial damages [59]. In addition, our data suggest that increasing HSP27 expression and phosphorylation in the heart by the combination of binge ethanol+MDMA is a biological strategy to minimize myocardial dysfunction induced by both drugs of abuse.

Our data also demonstrated that the cardiac changes in sympathetic pathways and the increase in HSP27 activity after the combination of both drugs are only evident 48 h after MDMA treatment. Additionally, 72 h or 7 days after treatment, ethanol-treated or MDMA-treated animals showed changes in these parameters but the combination of both drugs did not increase the activity in sympathetic pathway or the activation of HSP27 suggesting that the potential risk of the consumption of ethanol and MDMA in combination is more evident during a short period after concomitant exposure.

In conclusion, our results presented here clearly show that binge ethanol+MDMA association is capable of inducing the activation of cardiac sympathetic pathways in parallel with an enhancement of HSP27 expression and phosphorylation and suggests that this chaperone can protect the heart against cardiac changes observed after binge exposure to ethanol associated with MDMA, probably through its antiapoptotic and oxidative stress-attenuating properties.

## Abbreviations

MDMA: 3,4-Methylenedioxymethamphetamie
DID: drinking in the dark
TH: tyrosine hydroxylase
NA: noradrenaline
NMN: normetanephrine
HSP27: heat shock protein 27
