# Correction: Binge Ethanol and MDMA Combination Exacerbates Toxic Cardiac Effects by Inducing Cellular Stress

**DOI:** 10.1371/journal.pone.0143462

**Published:** 2015-11-16

**Authors:** Javier Navarro-Zaragoza, Clara Ros-Simó, María-Victoria Milanés, Olga Valverde, María-Luisa Laorden

In the Funding section, the grant number from the funder Ministerio de Economía y Competitividad is listed incorrectly. The correct grant number is: SAF/FEDER 2013-49076-P.
